# Transition of Metabolic Phenotypes and Risk of Atrial Fibrillation According to BMI: Kailuan Study

**DOI:** 10.3389/fcvm.2022.888062

**Published:** 2022-06-28

**Authors:** Maoxiang Zhao, Wenjuan Du, Qianqian Zhao, Yating Chen, Bin Li, Zhonghui Xie, Zihao Fu, Nan Zhang, Xiaowei Cheng, Xiaoqian Li, Siyu Yao, Miao Wang, Chi Wang, Shouling Wu, Hao Xue, Yang Li

**Affiliations:** ^1^Department of Cardiology, First Medical Center, Chinese People’s Liberation Army Hospital, Medical School of Chinese People’s Liberation Army, Beijing, China; ^2^Laboratory of Radiation Injury Treatment, Medical Innovation Research Division, PLA General Hospital, Beijing, China; ^3^Department of Cardiology, Fujian Medical University, Fuzhou, China; ^4^School of Medicine, Nankai University, Tianjin, China; ^5^Department of Cardiology, Kailuan Hospital, Tangshan, China; ^6^Department of Cardiology, Sixth Medical Center, Chinese People’s Liberation Army Hospital, Medical School of Chinese People’s Liberation Army, Beijing, China

**Keywords:** atrial fibrillation, obesity, metabolic health, transition, body mass index

## Abstract

**Objective:**

Atrial fibrillation (AF) is associated with both obesity and its metabolic consequences. However, there is a paucity of information on whether the dynamic change of metabolic health and obesity phenotypes affect the risk of AF. We aimed to prospectively examine the association between metabolic health and its change over time and AF risk across body mass index (BMI) categories.

**Methods:**

A total of 58,483 participants without history of cancer, and cardiovascular diseases from the Kailuan study were included in the present study. Transition of metabolic phenotypes was evaluated between 1st survey (2006–2007) and the 2nd survey (2008–2009). The hazard ratios (HRs) and 95% confidence intervals (CIs) for AF were assessed by Cox proportional hazards regression.

**Results:**

During a median follow-up of 3 years, we documented 580 cases of AF. Compared with metabolically healthy individuals with normal weight, the multivariable-adjusted hazard ratios for metabolically healthy and unhealthy overweight/obese were 1.27 (95% *CI*: 1.01, 1.59) and 1.37 (95% *CI*: 1.09, 1.72), respectively. However, when transition was taken into account, overweight/obese people who maintained metabolically healthy status were not associated with increased long-term risk (*HR*, 1.11;95% *CI*: 0.70, 1.78), whereas participants who converted from metabolically healthy overweight/obese status to an unhealthy phenotype had higher AF risk than those who maintained metabolically healthy normal weight (*HR* 1.59, 95% *CI*: 1.11, 2.26). When BMI and metabolically healthy status were updated over the course of the study, significant short-term elevations in AF risk were associated with individuals with stable MU-OW/OB status.

**Conclusion:**

In this community-based cohort study, metabolically healthy overweight/obese individuals have increased risks of AF. Obesity remains a risk factor for AF independent of major metabolic factors. Our data further suggested that metabolic phenotype was a dynamic condition, and maintenance of metabolic health and normal weight might alleviate the risk of AF.

## Introduction

The increasing prevalence of atrial fibrillation (AF) is a growing global epidemic concern afflicting more than 33 million people worldwide in 2010, with estimated 5 million new cases arising annually (1–5). If this rapid growth continues, a dramatic increment in the health care costs is expected (6). It is therefore, the need for greater understanding of modifiable risk factors and implementing new strategies of prevention to manage this severe condition is urgent.

Overweight and obese are well-known risk factors associated with occurrence of incident AF (7). Overweight and obesity have a negative impact on the development of AF, which is mediated in part by metabolic abnormalities such as hypertension, hypercholesterolemia, and hyperglycemia (1, 8). However, individuals with overweight and obese have variation in metabolic disorders. It has been reported that there is an unique subset of obese individuals having a relatively favorable metabolic profile, termed as metabolically healthy obese (MHO) (9, 10). Contrary to some previous observations that MHO do not entail an increased risk for cardiovascular diseases (CVDs) compared with normal weight metabolically healthy individuals (11–13), recent studies have suggested that MHO phenotype was not a benign condition and associated with higher risk of chronic diseases such as CVD (14–16). However, only limited data were available for the relationship between AF and MHO and the results were conflicting (17–19). The inconsistency may due to the ethic difference and ignoring the dynamic nature of metabolic health status. Recent studies reported that MHO is a dynamic condition that changes over time and this metabolic transition may alter the risk of metabolic complications (20–23). To date, the information regarding the effects of transitions in metabolic health across the obesity status on AF risk are lacking.

Therefore, the present study aimed to examine the associations of BMI categories and metabolic health status and their transition over time with AF in a large Chinese cohort study.

## Methods

### Study Population

**FIGURE 1:**
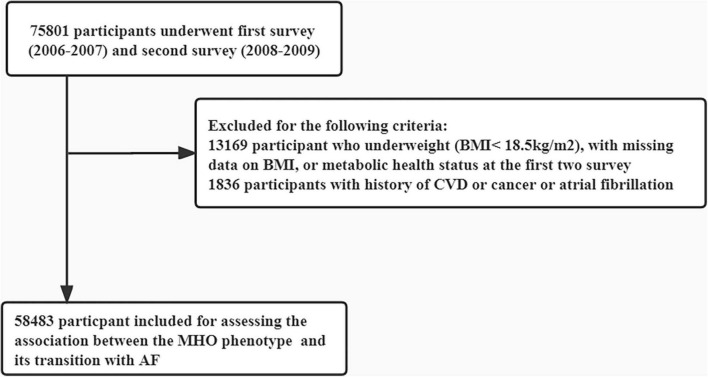
Flow chart of the current study.

The study population was obtained from the Kailuan Study, which is an ongoing cohort study conducted in a community-based population in Tangshan City, China. A detailed description of the study design and procedures has been published previously (24, 25). Briefly, 101,510 participants aged 18 years or above were enrolled between 2006 and 2007 at 11 hospitals affiliated with the Kailuan community. All of the participants were clinically follow-up approximately every 2 years to obtain information via face-to-face interviews with medical staff. For the present study, participants who underwent 1st (2006–2007) and 2nd (2008–2009) survey were included (*n* = 75,801). Of these remaining subjects, those with missing parameters (e.g., use of lipid-lowering, antidiabetic, or antihypertensive drugs, body mass index, HDL-C, triglycerides, blood pressure, history of diabetes and hypertension), or with previous history of cancer and CVD, or with physician diagnosed AF, or underweight were excluded. A total of 58,483 participants were included in the final analyses (Figure 1). This study was approved by the Ethics Committees of the Kailuan General Hospital), and informed consent was provided.

### Assessment of BMI Categories and Metabolic Health Status and Their Transition

Height and weight were measured by trained staffs while the participants were barefoot and in light clothing. Blood pressure was measured twice from the left arm with participants in a seated position after at least 15 min of rest, using a mercury sphygmomanometer. Venous blood samples were obtained and transfused into vacuum tubes containing EDTA in the morning after participants fasted for at least 8 h. Fasting blood glucose (FBG), triglyceride (TG), total cholesterol, low-density lipoprotein cholesterol (LDL-C), and high-density lipoprotein cholesterol (HDL-C) were measured using an auto-analyzer (Hitachi747; Hitachi, Tokyo, Japan). Serum levels of C-reactive protein (CRP) were determined by an immunoturbidimetry assay (Kanto Chemical Co Inc., Tokyo, Japan), with a lower limit of detection of 0.1 mg/L. High hs-CRP levels were defined as serum hs-CRP >3 mg/L.

We classified participants into BMI categories based on recommendations of the Working Group on Obesity in China (26): normal weight (BMI 18.5–23.9 kg/m^2^), overweight (BMI 24.0–27.9 kg/m^2^), and obese (BMI ≥ 28 kg/m^2^). Individuals with none or one of the following disorders were deemed metabolically healthy (27): (1) elevated SBP (≥130 mmHg) and/or DBP (≥85 mmHg), or the use of antihypertensive drugs, or self-reported physician-diagnosed hypertension; (2) high FPG (≥5.6 mmol/l), or the use of antidiabetic drugs, or self-reported physician-diagnosed diabetes; (3) low HDL-C (<1.04 mmol/l in men and <1.29 mmol/l in women); and (4) high triacylglycerols (≥1.7 mmol/l) or the use of lipid-lowering drugs.

Based on the combination of BMI categories and metabolic health status, participants were then categorized into four groups: metabolically healthy normal weight (MH-NW); metabolically healthy overweight/obese (MH-OW/OB); metabolically unhealthy normal weight (MU-NO); metabolically unhealthy overweight/obese (MU-OW/OB). Transition in the metabolic health across BMI categories were examined at the follow-up biannual health examination between 1st and 2nd survey. Transitions in metabolic health yielded six phenotypes: stable MH-NW; MH-NW to MU (metabolically unhealthy); MU-NW; stable MH-OW/OB; MH-OW/OB to MU-OW/OB; MU-OW/OB (Supplementary Table 1).

### Covariates

Information on age, sex, smoking status (yes or no), alcohol consumption (yes or no), and physical activity level (sedentary or active) were obtained via standardized questionnaires. Smoker was defined as those who had a history of smoking. Drinker was defined as those who had a history of drinking. Physical activity was classified into two categories: sedentary/active activity for aerobic exercise <3 times/week, and aerobic exercise ≥3 times/week.

### Outcome

The primary endpoint was newly diagnosed AF during the follow-up period. AF diagnoses were retrieved from discharge registers at the municipal social insurance that cover all of the Kailuan study participants and resting ECGs of each survey. At each biennial survey, a 12-lead electrocardiogram performed with participants in the supine position, after a 5-min or longer rest in the quite room. The ECG and diagnosis were completed by two professional electrocardiologists according to the European Society of Cardiology guideline (28).

### Statistical Analysis

Baseline characteristics are presented as mean ± SD for normally distributed data, or as median (inter quartile ranges) for not normally distributed data and n (%) for categorical variables. Comparisons were made using chi-square tests for categorical variables and one-way ANOVA test for continuous variables. Cox proportional hazards model was used to examine the relationship of different phenotypes and incident AF. The risk of incident AF was first analyzed according to the baseline metabolic health and BMI categories without considering their transition, with the MH-NW phenotype as the reference. Next, we further assessed the association between the transitions in metabolic health from 2006–2007 to 2010–2011 and the risk of AF incidence during the follow-up (after 2010–2011). All Cox proportional hazards regression models were adjusted for the following covariates: age, sex, physical activity, smoke status, drink status, LDL-C, and C-reactive protein. The proportional hazard assumptions for the main Cox models were tested and no violations have been observed. To investigate the relationship between the short term risk of transitions in metabolic health status and subsequent risk of incident AF, we also constructed time-varying Cox models where BMI categories and metabolic health status were updated at each follow up and the most recent BMI measurement was used to estimate risk in the following time period. In multivariable-adjusted models, other covariates were updated at various time points, and if data were missing at a given time point, the last observation was carried forward. As recommended by Knol and VanderWeele (29, 30), additive interaction of comorbidity and hip fracture was calculated based on the relative excess risk due to interaction (RERI) compared to no hip fracture and CCI = 0, while multiplicative interaction was based on the ratio of relative risks.

To assess the robustness of our findings, we performed several sensitivity analyses:

(1) We excluded outcome events occurring within the first year of follow-up to address the potential reverse causation; (2) We excluded the participants developing CVD during follow-up; (3) We further adjusted for the weight change. In time-varying cox model, weight changes were calculated for each subject as the differences in weight from visit 2 to baseline (visit 1), from visit 3 to visit 2, from visit 4 to visit 3, and in the same way afterward. In Cox proportional hazards model, weight change calculated as between baseline and 2nd survey; (4) We repeated our analyses of incident AF using Fine-Gray models instead, accounting for the competing risk of death; Moreover, we analyzed the data of participants who were initially eligible at first survey (2006–2007) to assessing the risk of incident atrial fibrillation cross-classified by metabolic health status and obesity at baseline. All statistical analyses were conducted using SAS 9.3 (SAS Institute, Cray, NC, United States). Tests were two-sided, and a *p*-value < 0.05 was considered statistically significant.

## Results

Table 1 presents the participants’ baseline characteristics stratified by BMI categories and metabolic health status. Among 58,483 participants, 34.3% (*n* = 20,083) and 24.1% (*n* = 14,080) were classified as MH-NW and MU-OW/OB, respectively.

**TABLE 1:** Characteristics of participants according to metabolic health and body mass index categories.

Baseline characteristics	Normal weight		Overweight or obese		
	Metabolically healthy (MH-NW)	Metabolically unhealthy (MU-NW)	Metabolically healthy (MH-OW/OB)	Metabolically unhealthy (MU-OW/OB)	*P-value*
No. of participants	20,083	7,915	13,506	14,080	
Age	48.12 ± 12.40	52.05 ± 10.99	48.41 ± 11.86	51.29 ± 10.76	<0.01
Male, *n* (%)	15,378 (76.57)	6,464 (81.67)	11,026 (81.64)	11,785 (83.70)	<0.01
Physical activity ≥ 3 times/week, n (%)	2,835 (14.16)	1,308 (16.68)	1,858 (13.80)	17.10 (17.10)	<0.01
Smoker, *n* (%)	8,282 (41.34)	3,409 (43.44)	5,375 (39.90)	5,808 (41.57)	<0.01
Drinker, *n* (%)	8,461 (42.21)	3,491 (44.50)	5,732 (42.52)	6,033 (43.16)	<0.01
BMI, kg/m^2^	22.43 ± 1.63	23.02 ± 1.49	27.50 ± 2.21	28.18 ± 2.51	<0.01
SBP, mmHg	120.99 ± 17.21	137.61 ± 18.44	126.81 ± 18.66	141.01 ± 18.94	<0.01
DBP, mmHg	78.36 ± 9.92	86.72 ± 10.48	82.38 ± 10.67	89.91 ± 11.17	<0.01
LDL, mmol/L	2.33 ± 0.86	2.43 ± 0.95	2.43 ± 0.85	2.48 ± 0.95	<0.01
HDL, mmol/L	1.58 ± 0.38	1.52 ± 0.44	1.53 ± 0.35	1.47 ± 0.41	<0.01
TG, mmol/L	1.14 ± 0.74	2.25 ± 1.81	1.38 ± 0.91	2.58 ± 1.74	<0.01
CRP, mg/L	0.50 (0.20, 1.40)	0.70 (0.26, 1.89)	0.83 (0.33, 2.00)	1.07 (0.45, 2.52)	<0.01

*BMI, body mass index; HDL, high-density lipoprotein; LDL, low-density lipoprotein; SBP, systolic blood pressure; DBP, diastolic blood pressure; CRP, C-reactive protein; TG, triglyceride. Values are presented as mean ± SD or median (25th–75th) or n (%).*

The risk of CVD for participants cross-classified by BMI categories and metabolic health were shown in Table 2. During 5.3 million person-years of follow-up, 580 patients with new-onset AF were identified. After adjustments for clinical variables, participants with MH-OW/OB and MU-OW/OB were associated with 1.27-fold (95% *CI*: 1.01, 1.59) and 1.37-fold (95% *CI*: 1.09, 1.72) increased AF risk, respectively, compared with participants with MH-NW. We found no significant multiplicative and additive interaction between BMI and metabolic healthiness [*p* for additive interaction > 0.05; RERI = 0.52 (95% *CI*: −0.48, 1.53); *p* for multiplicative interaction > 0.05].

**TABLE 2:** Hazard ratios for incident atrial fibrillation classified by weight categories and metabolic health status.

Obesity phenotype	Case/total	Rate	Multivariable adjusted HR (95% *CI*)*^a^*	HR (95% *CI*) in model using time-dependent variables*^b^*
MH-NW	136/15,908	0.74	1.00 (reference)	1.00 (reference)
MU-NW	41/5,377	0.69	0.89 (0.56,1.16)	1.36 (0.97, 1.91)
MH-OW/OB	190/17,803	0.92	1.27 (1.01, 1.59)	1.35 (1.08, 1.67)
MU-OW/OB	213/16,721	1.13	1.37 (1.09, 1.72)	1.63 (1.30, 2.05)

*^a^The multivariable model was adjusted for age, sex, physical activity, smoke status, drink status, LDL-C, and C-reactive protein.*

*^b^Model with age, sex as a time-fixed categorical variables, and physical activity, smoke status, drink status, LDL-C, and C-reactive protein as a time-dependent categorical variables.*

We next examined the dynamic association between BMI and AF by updating measures of BMI and metabolic health status, and participants with MH-OW/OB and MU-OW/OB were associated with a significant elevation in the risk of AF. Even after adjustment for updated measures of potential confounders, such as physical activity, smoke status, drink status, LDL-C, and C-reactive protein, MH-OW/OB and MU-OW/OB status remained significantly associated with short-term elevations in AF risk.

We further investigated the effect of changes in metabolic health status during follow-up. Among participants with metabolic health, 14.3% (*n* = 8,390) MH-NW and 21.2% (*n* = 12,402) MH-OW/OB converting to metabolically unhealthy phenotype. Compared with stable MH-NW individuals, overweight/obese participants who were metabolically unhealthy at baseline or transitioned to metabolically unhealthy phenotype were at increased risk of AF (*HR*: 1.59; 95% *CI*: 1.11, 1.26 for MH-OW/OB to MU; *HR*: 1.63; 95% *CI*: 1.16, 2.31 for MU-OW/OB). However, the long-term AF risk was not significantly elevated among stable MHO individuals after multivariable adjustment. In time-varying models introducing transition in metabolic health status, BMI, and confounders as time-varying covariates, participant who maintained stable MH-OW/OB and transitioned from MH-OW/OB to unhealthy metabolic status had 1.35-fold (95% *CI*: 1.06, 1.73) and 1.52-fold (95% *CI*: 1.18, 1.95) higher short-term risk of AF than participant with stable MH-NW, respectively (Table 3).

**TABLE 3:** Hazard ratios for incident atrial fibrillation according to maintenance or transition of phenotypes in MHO group.

Changes in metabolic health across weight categories	Case/total	Rate	Multivariable adjusted HR (95% *CI*)^a^	HR (95% *CI*)^b^ in model using time-dependent variables
MH-NW to MH	44/7,248	0.52	1.00 (reference)	1.00 (reference)
MH-NW to MU	88/8,390	0.92	1.16 (0.86, 1.85)	1.15 (0.66, 2.10)
MU-NW	41/5,377	0.68	1.01 (0.59, 1.44)	1.11 (0.76, 1.62)
MH-OW/OB to MH	33/5,086	0.55	1.11 (0.70, 1.78)	1.35 (1.06, 1.73)
MH-OW/OB to MU	154/12,402	1.08	1.59 (1.11, 2.26)	1.52 (1.18, 1.95)
MU-OW/OB	213/16,721	1.28	1.63 (1.16, 2.31)	1.79 (1.29, 2.50)

*^a^The multivariable model was adjusted for age, sex, physical activity, smoke status, drink status, LDL-C, and C-reactive protein.*

*^b^Model with age, sex as a time-fixed categorical variables, and physical activity, smoke status, drink status, LDL-C, changes in metabolic health and C-reactive protein as a time-dependent categorical variables.*

In the sensitivity analyses, the association between MHO phenotype and its transitions with AF development was not altered by excluding participants with AF occurring within the first years of the follow-up (Supplementary Tables 2, 3), or excluding the participants developing CVD during follow-up (Supplementary Tables 4, 5) or additional adjustment for weight change (Supplementary Table 6). To account for the competing risk of mortality, we conducted a competing risk model and the results remains similar to main results (Supplementary Tables 7, 8). Furthermore, we analyzed the data of participants who were initially eligible at first survey (2006–2007) to assessing the risk of incident atrial fibrillation cross-classified by metabolic health status and obesity at baseline (Supplementary Table 9).

## Discussion

In this large community-based cohort study, we explore the association between the metabolic health status and its transition over time and AF risk across the BMI categories. We found that the individuals with MH-OW/OB had higher risk of developing AF, compared with those with MH-NW. Our results further supported the notion that metabolic health was a transient state. Particularly, metabolic status converting from healthy to unhealthy further increased the risk of AF among overweight/obese individuals.

Overweight and obesity are well-established risk factor for CVDs (7). One hypothesis explains this negative association is that overweight and obesity are associated with elevations in cardiometabolic traits (31). Although observational data from some independent studies reported that obesity with metabolic healthy was not at an increased risk for cardiovascular complications (9, 32), the association between CVD risk and MHO phenotype is still controversial. However, scarce evidence is available regarding whether individuals with MH-OW/OB without an increased AF risk. To date, only four studies have investigated the associations between metabolically healthy obesity and AF risk, but the results were conflicting. The HUNT study and a Swedish study reported that compared with the MH-NW, those with healthy and unhealthy obesity associated with similar AF risk, which is consistent with our results (17, 19). On the contrary, results of French and Korean study shown that MHO was associated with a higher risk of incident AF, compared with the MH-NW (18, 33). The inconsistency may due to the lack of a consensus definition of “metabolically healthy” and the different specificity of an AF diagnosis. In our study, a stricter definition of metabolic health (an absence of all metabolic abnormalities) was adopted, an adverse effect of obesity or overweight with a metabolically healthy status on incident AF was revealed. This supports the notion that obesity and overweight itself was associated with the risk of incident AF independent of metabolic comorbidities.

More recent evidence suggested that MHO phenotype might be a transient in nature. The percentage of individuals converting from MHO to MUO was reported to vary between 30 and 50% during 4–10 years (11, 34–36). Besides, latest findings from the Nurses’ Health Study (NHS) and a Chinese study shown that over 80 and 50% of the participants with metabolically healthy obesity at baseline converted to a metabolically unhealthy phenotype during the follow-up (14, 22). In line with previous study, 23.1% original MH-OW/OB converted to metabolic unhealthy status over a period of 3 years. Thus, these findings imply that single point determination of metabolic health may be not sufficient to predict AF risk.

Several recent studies have reported that the transition from MHO to MUO increased the risk of adverse health outcomes, such as atherosclerosis, chronic kidney disease, and CVD events (14, 20, 35, 37–40). However, whether the transition of metabolic health status could affect the risk of AF development among different obesity status remains unknown. In the present study, we adopted two approaches to address the change in metabolic health status over time. First, we investigate the long-term effect of transition of metabolic health on AF development, by following up the metabolic health status. We found that having MH-OW/OB at baseline conferred high risk of AF on individuals who experienced a transition to metabolically unhealthy status later, but the participants remain stable MH-OW/OB had non-significant long-term risk of AF. Second, we utilized updated measures to account for changes in MHO phenotype over time and to characterize the short term impact MHO phenotype has on AF risk. Our results utilizing updated BMI and metabolic health status are consistent with data from other population based cohort studies utilizing single time point determination of metabolic health status (18, 33). MH-OW/OB and MU-OW/OB were at higher AF risk. In addition, individuals with stable MH-OW/OB was associated with significant short-term elevations in AF risk, compared with persistent MH-NO.

Although the exact causal mechanism has not been completely understood, the present findings are biologically plausible. Accumulating evidence suggests that individuals who are initially metabolically unhealthy or convert so during follow-up have less adequate subcutaneous adipose tissue expansion, leading to less subcutaneous, more visceral fat mass, and a higher ectopic fat deposition in the liver than those who remained metabolically healthy (41–44), which have been associated with higher levels of inflammation, insulin resistance. All these pathophysiological changes were the major triggers for CVDs, which may result in atrial fibrillation (45–49).

The present study expanded the current body of knowledge on the impact of MHO phenotype on risk of AF. Several findings from the current analysis bear mention. First, results of our study suggested that overweight/obese was associated with AF risk regardless of metabolic status. Second, we confirmed that MHO phenotype was a dynamic condition and participants with overweight/obese converting from metabolically healthy to unhealthy status further increased the risk of AF. In addition, we also revealed that conversion rate from metabolically healthy to metabolically unhealthy status in overweight/obesity individuals was higher than normal weight individuals, which may imply that obesity is an accelerating factor for metabolic abnormalities. Therefore, findings of our study support the public health policy that early management of body weight should be encouraged for the public and with aim to prevent the development of metabolic syndrome, which can be accomplished by physical activity, exercise training, and incorporating dietary strategies of reduced caloric intake (50).

Strengths of the present study include its prospective design, large sample size, updated measures of MHO phenotype, and long-term follow-up. Moreover, to the best of our knowledge, this is the first study to explore the association between transition of MHO phenotype and AF risk. Several limitations to our work should also be acknowledged. First, as the study population consisted of only Chinese, the results may not be generalizable to other ethnicities. Second, detailed data regarding the subtypes of AF, which may or may not have influenced the future development of AF, were not collected. Third, although we adjusted for covariates with either known or suspected relationships with AF risk, residual confounding always remains a possibility. Fourth, given the observational nature of the present study, the finding is not sufficient for a causal relationship. Fifth, detailed data on the potential comorbidities such as COPD OSAHS, which may have influenced the future development of AF, were also not collected.

## Conclusion

In the present study, we suggested that overweight and obesity remains the risk factors of AF, regardless of the metabolic health status. Moreover, with repeated-measured of MHO phenotype, significant elevations in AF risk persisted in MH-OW/OB and its transition to a metabolically unhealthy status. Our findings highlight the need to maintain healthy weight and metabolic status.

## Data Availability Statement

The data that support the findings of this study are available from Kailuan study but restrictions apply to the availability of these data, which were used under license for the current study, and so are not publicly available. Data are however available from the corresponding author upon reasonable request and with permission of corresponding author.

## Ethics Statement

This study was approved by the Ethics Committees of Kailuan General Hospital. The patients/participants provided their written informed consent to participate in this study.

## Author Contributions

YL, HX, SW, and MZ designed the research. SW and MZ conducted the research. MZ and WD wrote the manuscript. MZ and CW analyzed the data. SY, QZ, YC, BL, ZX, ZF, NZ, XC, MW, and XL edited the manuscript. YL and MZ had primary responsibility for final content. All authors read and approved the final manuscript.

## Conflict of Interest

The authors declare that the research was conducted in the absence of any commercial or financial relationships that could be construed as a potential conflict of interest.

## Publisher’s Note

All claims expressed in this article are solely those of the authors and do not necessarily represent those of their affiliated organizations, or those of the publisher, the editors and the reviewers. Any product that may be evaluated in this article, or claim that may be made by its manufacturer, is not guaranteed or endorsed by the publisher.
