# Supplementary material for: Transition of Metabolic Phenotypes and Risk of Atrial Fibrillation According to BMI: Kailuan Study
Source: Front Cardiovasc Med. 2022 Jun 28;9:888062. doi: 10.3389/fcvm.2022.888062 (PMC9274110; doi:10.3389/fcvm.2022.888062)
Supplement: Supplementary file 1 [file Data_Sheet_1.doc]

**Supplemental Material**

**Table S1.** Definition of metabolic health phenotypes

**Table S2.** Hazard ratios and 95% confidence intervals for incident atrial fibrillation cross-classified by metabolic health and obesity: **Sensitivity analysis excluding outcome events within the first year of follow-up**

**Table S3.** Hazard ratios and 95% confidence intervals for incident atrial fibrillation according to maintenance or transition of phenotypes in MHO group: **Sensitivity analysis excluding outcome events within the first year of follow-up**

**Table S4.** Hazard ratios and 95% confidence intervals for incident atrial fibrillation cross-classified by metabolic health and obesity: **Sensitivity analysis excluding the participants developing CVD during follow-up**

**Table S5.** Hazard ratios and 95% confidence intervals for incident atrial fibrillation according to maintenance or transition of phenotypes in MHO group: **Sensitivity analysis excluding the participants developing CVD during follow-up**

**Table S6.** Hazard ratios and 95% confidence intervals for incident atrial fibrillation according to maintenance or transition of phenotypes in MHO group: **Sensitivity analysis additional adjustment for weight changes**

**Table S7.** Hazard ratios and 95% confidence intervals for incident atrial fibrillation cross-classified by metabolic health and obesity: **Competing risk analyses**

**Table S8.** Hazard ratios and 95% confidence intervals for incident atrial fibrillation according to maintenance or transition of phenotypes in MHO group: **Competing risk analyses**

**Table S9.** Hazard ratios and 95% confidence intervals for incident atrial fibrillation cross-classified by metabolic health and obesity: **Sensitivity analysis analyzing the data of participants who were initially eligible at first survey (2006-2007)**

**Table S1. Definition of metabolic health phenotypes**

| Metabolically healthy obese phenotype | Definition |
| --- | --- |
| MH-NW | BMI <24 kg/m^2^ and metabolically healthy status |
| MU-NW | BMI ≥24 kg/m^2^ and metabolically unhealthy status |
| MH-OW/OB | BMI ≥24 kg/m^2^ and metabolically healthy  status |
| MU-OW/OB | BMI ≥24 kg/m^2^ and metabolically unhealthy status |
| Stable MH-NW | Remain MH-NW at 1st and 2nd survey |
| MH-NW to MU | MH-NW at 1st and became metabolically unhealthy at 2nd survey |
| Stable MH-OW/OB | Remain MH-NW at 1st and 2nd survey |
| MH-OW/OB to MU | MH-OW/OB at 1st and became metabolically unhealthy at 2nd survey |

Abbreviations: metabolically healthy normal weight (MH-NW); metabolically unhealthy normal weight (MU-NW); metabolically healthy overweight/obese (MH-OW/OB); metabolically unhealthy overweight /obese (MU-OW/OB)

**Table S2.** Hazard ratios and 95% confidence intervals for incident atrial fibrillation cross-classified by metabolic health and obesity: **Sensitivity analysis excluding outcome events within the first year of follow-up**

| Obesity phenotype | Hazard ration (95%CI) |
| --- | --- |
| MH-NW | 1.00 (reference) |
| MU-NW | 0.79 (0.53, 1.17) |
| MH-OW/OB | 1.31 (1.02, 1.67) |
| MU-OW/OB | 1.44 (1.13, 1.83) |

Model was adjusted for age, sex, physical activity, smoke status, drink status, LDL-C, and C-reactive protein.

**Table S3.** Hazard ratios and 95% confidence intervals for incident atrial fibrillation according to maintenance or transition of phenotypes in MHO group: **Sensitivity analysis excluding outcome events within the first year of follow-up**

| Changes in metabolic  health across weight  categories | Hazard ration(95%CI) |
| --- | --- |
| MH-NW to MH | 1.00 (reference) |
| MH-NW to MU | 1.14 (0.89, 2.01) |
| MU-NW | 0.98 (0.59, 1.52) |
| MH-OW/OB to MH | 1.21 (0.74, 1.98) |
| MH-OW/OB to MU | 1.73 (1.18, 2.53) |
| MU-OW/OB | 1.81 (1.25, 2.62) |

Model was adjusted for age, sex, physical activity, smoke status, drink status, LDL-C, and C-reactive protein.

**Table S4. Hazard ratios and 95% confidence intervals for incident atrial fibrillation cross-classified by metabolic health and obesity: Sensitivity analysis excluding the participants developing CVD during follow-up**

| Obesity phenotype | Hazard ration (95%CI) |
| --- | --- |
| MH-NW | 1.00 (reference) |
| MU-NW | 0.77 (0.53, 1.11) |
| MH-OW/OB | 1.26 (1.01, 1.60) |
| MU-OW/OB | 1.45 (1.15, 1.83) |

Model was adjusted for age, sex, physical activity, smoke status, drink status, LDL-C, and C-reactive protein.

**Table S5.** Hazard ratios and 95% confidence intervals for incident atrial fibrillation according to maintenance or transition of phenotypes in MHO group: **Sensitivity analysis excluding the participants developing CVD during follow-up**

| Changes in metabolic  health across weight  categories | Hazard ration(95%CI) |
| --- | --- |
| MH-NW to MH | 1.00 (reference) |
| MH-NW to MU | 1.25 (0.85, 1.85) |
| MU-NW | 0.91 (0.58, 1.44) |
| MH-OW/OB to MH | 0.99 (0.60, 1.60) |
| MH-OW/OB to MU | 1.65 (1.15, 2.36) |
| MU-OW/OB | 1.71 (1.20, 2.43) |

Model was adjusted for age, sex, physical activity, smoke status, drink status, LDL-C, and C-reactive protein.

**Table S6. Hazard ratios and 95% confidence intervals for incident atrial fibrillation according to maintenance or transition of phenotypes in MHO group: Sensitivity analysis additional adjustment for weight changes**

| Changes in metabolic  health across weight  categories | Multivariable Adjusted HR  (95% CI)^a^ | HR (95% CI)^b^ in Model Using Time-Dependent Variables |
| --- | --- | --- |
| MH-NW to MH | 1.00 (reference) | 1.00 (reference) |
| MH-NW to MU | 1.15 (0.86, 1.84) | 1.18 (0.68, 2.06) |
| MU-NW | 0.99 (0.59, 1.44) | 1.11 (0.76, 1.62) |
| MH-OW/OB to MH | 1.13 (0.71, 1.81) | 1.38 (1.08, 1.77) |
| MH-OW/OB to MU | 1.60 (1.12, 2.29) | 1.54 (1.19, 1.98) |
| MU-OW/OB | 1.66 (1.17, 2.34) | 1.76 (1.19, 2.49) |

^a^The multivariable model was adjusted for age, sex, physical activity, smoke status, drink status, LDL-C, and C-reactive protein.

^b^Model with age, sex as a time-fixed categorical variables, and physical activity, smoke status, drink status, LDL-C, changes in metabolic health and C-reactive protein as a time-dependent categorical variables.

Model2 was further adjusted for weight change.

**Table S7.** Hazard ratios and 95% confidence intervals for incident atrial fibrillation cross-classified by metabolic health and obesity: **Competing risk analyses**

| Obesity phenotype | Hazard ration(95%CI) |
| --- | --- |
| MH-NW | 1.00 (reference) |
| MU-NW | 0.87 (0.55, 1.14) |
| MH-OW/OB | 1.29 (1.02, 1.63) |
| MU-OW/OB | 1.37 (1.09, 1.72) |

Model was adjusted for age, sex, physical activity, smoke status, drink status, LDL-C, and C-reactive protein.

**Table S8.** Hazard ratios and 95% confidence intervals for incident atrial fibrillation according to maintenance or transition of phenotypes in MHO group: **Competing risk analyses**

| Changes in metabolic  health across weight  categories | Hazard ration(95%CI) |
| --- | --- |
| MH-NW to MH | 1.00 (reference) |
| MH-NW to MU | 1.21 (0.88, 1.94) |
| MU-NW | 0.97 (0.61, 1.52) |
| MH-OW/OB to MH | 1.12 (0.69, 1.81) |
| MH-OW/OB to MU | 1.66 (1.17, 2.37) |
| MU-OW/OB | 1.73 (1.20, 2.48) |

Model was adjusted for age, sex, physical activity, smoke status, drink status, LDL-C, and C-reactive protein.

**Table S9.** Hazard ratios and 95% confidence intervals for incident atrial fibrillation cross-classified by metabolic health and obesity: **Sensitivity analysis analyzing the data of participants who were initially eligible at first survey (2006-2007)**

| Obesity phenotype | Hazard ration (95%CI) |
| --- | --- |
| MH-NW | 1.00 (reference) |
| MU-NW | 0.90 (0.65, 1.24) |
| MH-OW/OB | 1.29 (1.04, 1.60) |
| MU-OW/OB | 1.38 (1.12, 1.70) |

Model was adjusted for age, sex, physical activity, smoke status, drink status, LDL-C, and C-reactive protein.
